# Factors Affecting Intention of Consumers in Using Face Recognition Payment in Offline Markets: An Acceptance Model for Future Payment Service

**DOI:** 10.3389/fpsyg.2022.830152

**Published:** 2022-03-17

**Authors:** Dongyan Nan, Yerin Kim, Jintao Huang, Hae Sun Jung, Jang Hyun Kim

**Affiliations:** ^1^Department of Human-Artificial Intelligence Interaction, Sungkyunkwan University, Seoul, South Korea; ^2^Department of Interaction Science, Sungkyunkwan University, Seoul, South Korea; ^3^Department of Applied Artificial Intelligence, Sungkyunkwan University, Seoul, South Korea

**Keywords:** face recognition payment, financial technology adoption, perceived risk, relative advantage, trust

## Abstract

Face recognition payment (FRP), an innovative financial technology service, is a recently developed mode of payment service that has garnered attention in the offline market, particularly in China. However, studies examining the adoption of FRP by consumers are scarce. Therefore, this study proposed a causal model built on the Unified Theory of Acceptance and Use of Technology, and key predictors related to the intention of using FRP were identified. The structural equation model-based results obtained from 305 Chinese participants demonstrated that the intention was most affected by relative advantage. In addition, performance expectancy, effort expectancy, social influence, and perceived risk also had a significant impact. However, trust was found to not significantly affect consumers’ intentions, despite it negatively influencing perceived risk. Thus, the results of this study are expected to provide a set of guidelines for companies regarding the implementation of FRP.

## Introduction

Coupled with the development of financial technology (Fintech), innovative payment services have had a significant impact in changing the payment behavior of Chinese consumers. As of 2019, two representative third-party payment companies (i.e., Alipay and WeChat Pay) in China occupy 90% of the Chinese payment market (Li J. et al., 2019), and due to their rapid development, China has witnessed an acceleration in its transition to a “cashless society.”

With an aim to provide a better and improved service to consumers, Alipay and WeChat Pay launched a new payment system, i.e., a face recognition payment (FRP) called “Qing Ting” and “Qing Wa” in 2018 and 2019, respectively. This payment service is based on face recognition technology wherein a person is verified through a comparison of his/her facial features with faces existing within a database. Moreover, this technology has achieved an accuracy of over 99% (Lahasan et al., 2019).

In contrast to smartphone-based FRP (e.g., Apple Wallet’s facial ID and Samsung Pay’s facial ID), the FRP described in this study focuses solely on offline store payments. When attempting to make payments in an offline store, consumers need only scan their faces standing in front of the point-of-sale devices equipped with cameras. Moreover, no involvement of any mobile device (e.g., smartphone) renders it distinct from previous FRP options.

The incomparable advantage that FRP offers is its intuitiveness. Moreover, eliminating the need for mobile devices also facilitates a new sense of cashless society wherein the users need not have any sort of devices to make payments. Therefore, FRP can potentially replace the existing smartphone-based face recognition and other payment services (e.g., near-field communication and quick response), particularly in offline settings.

Although FRP service is still in its initial stage of development, several offline markets (e.g., supermarket, restaurant, fashion store, and hotel) in different cities around China have adopted them. Moreover, Alipay and WeChat Pay have announced their intention to invest 3 and 10 billion Yuan, respectively, with an aim to comprehensively promote the use of FRP services in offline markets (NetEase, 2019).

Consequently, FRP has garnered attention in academia as well. Although several studies related to FRP have been conducted (Abudarham et al., 2019; Dong et al., 2019; Li D. et al., 2019; Zhang et al., 2019; Zhi and Liu, 2019), they have mainly focused on the technical aspects of FRP, such as improving accuracy and security of face recognition technology. In contrast, studies related to FRP adoption are scarce.

In addition to the technical aspects of new products and services, the perspectives of individuals regarding their usage are crucial, i.e., in terms of success and diffusion of innovative products and services, the determinants that can affect the consumers’ usage intentions should be examined (Nan et al., 2020). Thus, this study attempted to examine the factors influencing consumers’ intentions regarding the use of FRP.

Therefore, a research model for the use of FRP based on the Unified Theory of Acceptance and Use of Technology (UTAUT) was constructed in this study. UTAUT is among the most well-known models related to technology adoption (Venkatesh et al., 2003; Nan et al., 2020), and it concurs that performance expectancy, effort expectancy, and social influence are positively and notably related to individuals’ intention to use particular technologies (Venkatesh et al., 2003). In addition, to comprehensively understand the use of FRP, the concepts of relative advantage, perceived risk, and trust were employed as key predictors of intention based on prior studies and Chinese Fintech development.

Relative advantage is a key factor in innovation diffusion theory (Rogers, 2003), which confirms its notable and positive effect on an individual’s intention to use specific innovative technologies (Rogers, 2003; Johnson et al., 2018; Yuen et al., 2018). Moreover, as for China’s Fintech development, several Fintech services (e.g., quick response code payment) have garnered attention in the offline market. However, the consumers must be able to perceive the advantages of FRP over existing Fintech services, or they will continue to use existing Fintech services instead of FRP. Therefore, this study attempts to explore the role of relative advantage in FRP adoption.

Although the security of Chinese e-finance witnessed drastic growth in the 2010s, several financial frauds and privacy violations still take place. This indicates that researchers should consider the consumers’ perceptions of the security of Fintech services in Chinese markets. In addition, Pavlou (2003); Khalilzadeh et al. (2017), and Shao et al. (2019) emphasized the role of the security-related factors such as perceived risk and trust in Fintech adoption. Moreover, Slade et al. (2015) and Al-Saedi et al. (2020) implied that perceived risk and trust concepts should be considered in the UTAUT model for certain Fintech services, which may provide a comprehensive understanding of consumer experiences. Particularly when using FRP, consumers need to provide their facial feature information, which may lead them to be more concerned about whether the FRP is risky and the service provider is trustworthy in the protection of their privacy and assets. Therefore, it was believed that incorporating the perceived risk and trust into the research model will facilitate to better understand FRP adoption.

Moreover, despite the importance of these three factors, existing studies on individual adoption of FRP in offline stores (Zhang and Kang, 2019; Zhong et al., 2021) have not considered them. Thus, in terms of its attempt to extend the discourse using UTAUT with relative advantage, perceived risk, and trust, this research can be considered meaningful.

## Literature Review

### Face Recognition Payment

The FRP, a biometric payment service, is based on face recognition technology. In China, this technology is utilized in both payment and transportation services. For example, starting from December 3, 2019, the city of Zhengzhou commercialized the technology in subway stations (South China Morning Post, 2019). Moreover, the FRP service has gained users outside China as well. In October 2019, Shinsegae Duty Free introduced WeChat Pay’s FRP service at approximately 40 major locations including Myeong-dong and Incheon International Airport (The Moodie Davitt Report, 2019) in South Korea. In addition, there have been attempts to start local FRP services as well. In 2019, South Korean Card company “Shinhan Card” launched its own FRP service called “Face pay,” (The Korea Times, 2020). Consequently, due to increasing popularity, several scholars have explored methods attempting to improve the technical aspects of FRP and related technologies (Dong et al., 2019; Li D. et al., 2019; Zhi and Liu, 2019). For instance, Zhi and Liu (2019) provided a face recognition model built on a genetic algorithm, which achieved an accuracy of approximately 99%. The ongoing academic discourse on FRP and related technologies implies that this technology is expected to be applied to more diverse fields and that FRP services are to be widespread in the offline market.

### Fintech Service Adoption

With the emergence of Fintech, several studies have attempted to explore consumers’ perspectives or intentions in the context of using Fintech services. Most have utilized Technology Acceptance Model (TAM) or UTAUT to construct models for the use of Fintech. Certain representative studies are as follows.

Khalilzadeh et al. (2017) constructed a UTAUT-based model and collected the data from 412 participants in the United States. Subsequently, based on the Structural Equation Modeling (SEM) results, they concluded that performance expectancy strongly and notably influenced the intention to use near-field communication payment in a restaurant. Hassan and Wood (2020) extended TAM with three variables (i.e., social influence, perceived risk, and trust) and explored the main factors affecting individuals’ intentions to utilize mobile banking. Lee et al. (2019a) constructed a TAM-based model and explored both consumers’ and retailers’ perspectives of mobile payment by examining the validated data of participating Koreans (304 consumers and 175 retailers). Patil et al. (2020) indicated that both effort expectancy and trust significantly influenced the intentions of Indians to adopt mobile payments employing a UTAUT-based model. Alalwan et al. (2017) analyzed the survey-based data from 343 Jordanians and found that performance expectancy, effort expectancy, trust, and hedonic motivation influenced the adoption of mobile banking services.

These studies indicate that the consumers’ intentions should be investigated to facilitate the diffusion of Fintech services (Nan et al., 2020). Moreover, Kim J.H. et al. (2021) has reported that exploring consumers’ perceptions or intentions in the context of specific innovative services is crucial to the success and diffusion of the innovative services. In addition, they found that most studies regarding Fintech have only explored the factors influencing the adoption of mobile payments or mobile banking. In other words, FRP adoption has been rarely discussed in the academic field. Thus, to contribute to the rapid diffusion of FRP, this study attempted to construct a UTAUT-based model and explore the adoption of FRP.

### Dependent Variable: Intention to Use

Intention refers to the subjective probability regarding how an individual consciously acts (or does not act) in a particular way (Warshaw and Davis, 1985; Shaw and Sergueeva, 2018). Thus, in this study, “intention to use FRP” was conceptualized as “*the subjective probability that individuals will consciously use or not use FRP*.” Due to the fact that the intention to use is strongly and notably related to the actual usage (Venkatesh et al., 2003), many user-oriented studies of particular technologies have employed intention to use as a dependent variable and explored the predictors that notably affected the intention to use (Shaw and Sergueeva, 2018; Nan et al., 2022).

### Unified Theory of Acceptance and Use of Technology

As an extension of TAM (Venkatesh et al., 2003; Zhou et al., 2010), UTAUT is a representative model for analyzing new technology adoption. With respect to the Fintech service, several empirical studies have constructed the UTAUT-based model and illustrated a high percentage of variance in intention (Baptista and Oliveira, 2015; Khalilzadeh et al., 2017; Patil et al., 2020). Venkatesh et al. (2003) reported that the intentions of consumers are mainly affected by performance expectancy, effort expectancy, and social influence.

Furthermore, although a facilitating condition is a variable within the UTAUT model, it was not considered in this study. This is because the facilitating condition is related to the actual usage of particular technologies, rather than the intention to use them (Venkatesh et al., 2003; Khalilzadeh et al., 2017).

#### Performance Expectancy

The term “performance expectancy” has been considered as one of the core predictors in UTAUT. In this study, “performance expectancy” was conceptualized as “*the level to which consumers think that FRP is useful and beneficial to them*” (Venkatesh et al., 2003). With respect to innovative products and services, several studies have demonstrated a strong association between performance expectancy and intention. Sohn and Kwon (2020) analyzed valid data from 378 Korean participants and demonstrated a positive effect of performance expectancy on the intentions of individuals to adopt artificial intelligence-based products. Regarding mobile payments, Lee et al. (2019b) reported that performance expectancy notably affected Korean consumers’ intentions. Consequently, with respect to FRP, this study hypothesizes that:

H1: Increased performance expectancy results in greater consumers’ intentions to use FRP.

#### Effort Expectancy

With respect to FRP, this study conceptualized “effort expectancy” as “*the level to which consumers think that using FRP does not need much effort*” (Venkatesh et al., 2003). Several scholars have concluded that effort expectancy can influence intention in a positive direction. In the case of information technology, Kabra et al. (2017) demonstrated a notable association between effort expectancy and intention by examining the survey-based data from 192 participants. Liébana-Cabanillas et al. (2019) analyzed the data from 180 Spanish consumers and reported that effort expectancy exerted a positive influence on the intention to adopt NFC payments in public transportation. However, this connection is not always notable. For instance, as individuals gain more knowledge using particular systems, they believe that they can utilize the systems proficiently (Hackbarth et al., 2003) and, consequently, may not be bothered about the ease of using the systems (Slade et al., 2015). Thus, effort expectancy is likely not to affect intention to use to a significant level in this case.

Moreover, if consumers perceive using a specific technology does not require much effort, they are bound to consider the technology as useful and beneficial to them (Davis, 1989; Khalilzadeh et al., 2017). Thus, performance expectancy can act as the mediator in the relationship between effort expectancy and intention. This association has also been validated by several studies. Oliveira et al. (2016) verified that effort expectancy indirectly affected the intention to adopt mobile payments *via* the mediation of performance expectancy based on the SEM results obtained from 301 participants. Regarding mobile banking, Alalwan et al. (2017) concluded that effort expectancy can indirectly impact intention *via* the mediation of performance expectancy by examining the data from 348 consumers. Thus, with respect to FRP, this study hypothesized that:

H2: Increased effort expectancy results in greater performance expectancy of FRP.

H3: Increased effort expectancy results in greater consumers’ intentions to use FRP.

#### Social Influence

With respect to FRP, this study conceptualized “social influence” as “*the level to which individuals feel that their important acquaintances think they should use FRP*” (Venkatesh et al., 2003). Certain empirical studies have demonstrated a remarkable connection between social influence and intention. Chiyangwa and Alexander (2016) found that social influence significantly affected the intention to use multimedia message services by analyzing the data obtained from South Africa. Furthermore, Lin et al. (2019) examined data from 908 participants (467 Chinese and 441 Koreans) and confirmed the positive influence of social influence on both Chinese’ and Koreans’ intentions to use mobile payments. Thus, with respect to FRP, this study hypothesized that:

H4: Increased social influence results in greater consumers’ intentions to use FRP.

### Perceived Risk and Trust

When considering FRP, “perceived risk” is interpreted as “*the level to which consumers feel uncertain or anxious about the negative outcomes of using FRP*” (Phonthanukitithaworn et al., 2015). Liu et al. (2021) stated that if consumers feel insecure about their financial assets and privacy when considering certain services, they may refuse to use them. In addition, prior studies (Brown and Deegan, 1998; Cao and Niu, 2019) have implied that negative media coverage of a particular technological product and service can cause the public to have a negative perception of and to refuse using them. Considering these perspectives, earlier studies have verified that perceived risk can reduce intention to utilize certain Fintech services. For instance, Shao et al. (2019) demonstrated that the intention to adopt mobile payment can be inversely predicted based on the perceived risk, by analyzing the usable data from 740 Chinese respondents. Moreover, Kalinic et al. (2019) verified that perceived risk can influence the intention to utilize mobile payment in a negative direction by examining the valid data from 701 respondents from Spain. In addition, this association also has been verified and supported in the autonomous vehicle area (Lee et al., 2019c). Therefore, with respect to FRP, this study hypothesized that:

H5: Increased perceived risk results in weaker consumers’ intentions to use FRP.

This research interprets “trust” as “*the level to which consumers believe that FRP service providers will perform certain activities for meeting consumers’ expectations*” (Shin, 2009). Alalwan et al. (2017) mentioned that if individuals believe the service providers to be trustworthy, they may be motivated to use specific services. In addition, several studies have found a notable connection between trust and intention (Zhou, 2012; Shao et al., 2019). For example, Zhou (2012) reported that trust can increase Chinese consumers’ intentions to use mobile banking.

Moreover, trust can be used to indirectly predict the consumers’ intentions through the mediation of perceived risk (Pavlou, 2003) and can also reduce consumers’ anxiety regarding the negative outcomes of using payment services to purchase products (Hwang et al., 2014). With respect to Fintech service, earlier studies have demonstrated the role of trust in reducing perceived risk (Zhao et al., 2010; Slade et al., 2015; Shao et al., 2019). For instance, regarding internet banking, Zhao et al. (2010) concluded that perceived risk can be reduced by “trust” by analyzing the usable data for 432 participants in China. In addition, Shao et al. (2019) demonstrated that trust can influence the perceived risk of mobile payments in a negative direction. Therefore, with respect to FRP, this study hypothesized that:

H6: Increased trust results in greater consumers’ intentions to use FRP.

H7: Increased trust results in weaker perceived risk of FRP.

### Relative Advantage

With respect to FRP, this study conceptualized relative advantage as “*the level to which consumers feel that FRP can provide more benefits than its predecessors*” (Rogers and Shoemaker, 1983). Innovation diffusion theory concurs that relative advantage is a core predictor that results in a stronger intention to utilize specific technologies (Rogers, 2003). Moreover, this connection has been validated in certain research related to innovative products and services. Yuen et al. (2018) examined the valid data obtained from 164 Singaporeans and found that relative advantage significantly affected the consumers’ intentions to utilize self-collection services in a positive direction. Furthermore, regarding mobile payment, Johnson et al. (2018) discovered a notable connection between relative advantage and intention based on the SEM results obtained from 270 respondents.

Due to the similar conceptualizations of relative advantage and performance expectancy (refer to the section “Performance Expectancy”), a significant connection between these two factors is expected. Similar to the perspective of Min et al. (2019), consumers are expected to evaluate the FRP’s relative advantage through comparisons with prior payment services that they have used. This helps them better understand the benefits of FRP. In addition, this association has been demonstrated and supported in the context of smart wearable technology (Kim and Shin, 2015), Haptic Enabling Technology (Oh and Yoon, 2014), and Uber mobile application (Min et al., 2019). Considering that the performance expectancy can act as a mediator in influencing individuals’ intentions (Alalwan et al., 2017), the abovementioned perspectives also imply that relative advantage can positively impact intention to use *via* the mediation of performance expectancy. Thus, with respect to FRP, this study hypothesized that:

H8: Increased relative advantage results in greater performance expectancy of FRP.

H9: Increased relative advantage results in greater consumers’ intentions to use FRP.

## Materials and Methods

All the survey items employed in this study have been validated in earlier research (Appendix). Furthermore, the scales of UTAUT and intention to use were chosen according to the study by Venkatesh et al. (2003), whereas the scales used for perceived risk and trust were those reported by Featherman and Pavlou (2003) and Jarvenpaa et al. (1999), respectively. In addition, the relative advantage scale was acquired from the study by Kim et al. (2009). Finally, the original questionnaire items were appropriately revised to reflect the theme of FRP.

Based on Lien et al.’s (2017) suggestions, this study conducted an online survey on Sojump (i.e., a platform similar to SurveyMonkey), which is a representative online survey platform in China. Consequently, the usable data of 305 Chinese respondents (140 men and 165 women) were gathered. The age variation of the participants was as follows. The age group of 18–29 years accounted for 46.88% of the participants while those in the age group of 30–39 years were 36.72%. Furthermore, participants in the age group of 40–49 years and 50 + years constituted 8.2% of the total respondents. Moreover, approximately 28% of participants had the experience of using FRP in offline stores.

According to NetEase (2021), there are about 243 million FRP users in China, accounting for roughly 23% of the Chinese population over the age of 18 years (Statista, 2021a,b). In addition, Zhong et al. (2021) implied that consumers aged 18–40 years account for more than 80% of the actual and potential users of FRP in China. Therefore, this study reports that the sample composition does not deviate from the current proportion of FRP users among the whole Chinese population aged 18 years or above.

To ensure that all the respondents are fully aware of how this FRP service functions, a brief introduction of the FRP and a short demo video explaining the use of FRP were included in the survey procedure. The video showcased a consumer completing the payment *via* scanning his face in front of a point-of-sale machine equipped with a camera, at an offline store. All the survey items used a 5-point Likert scale (from 1 = “Strongly disagree” to 5 = “Strongly agree”) (Nan et al., 2020; Kim Y. et al., 2021).

## Results

### Validity

Pertaining to the suggestions of prior studies (Mariani et al., 2021; Chang and Lee, 2022; Nan et al., 2022), this research computed factor loading, average variance extracted (AVE), and composite reliability by performing confirmatory factor analysis with AMOS 23. Additionally, Cronbach’s alpha was computed with SPSS 27. As shown in Table 1, the outcomes indicated that all the values had surpassed the recommended levels (Cronbach’s alpha > 0.7, Factor loading > 0.7, AVE > 0.5, and composite reliability > 0.7), which is suggested by Nunnally (1978) and Fornell and Larcker (1981). Furthermore, every square root value of the AVE was found to be higher than the inter-construct correlation values (Anderson and Gerbing, 1988; Table 2). Thus, the analyzed variables possess satisfactory psychometric properties.

**TABLE 1:** Validity and reliability.

Constructs	Items	Cronbach’s alpha	Factor loading	Average variance extracted	Composite reliability
Performance expectancy (PE)	PE1	0.896	0.862	0.739	0.894
	PE2		0.844		
	PE3		0.872		
Effort expectancy (EE)	EE1	0.916	0.847	0.788	0.917
	EE2		0.921		
	EE3		0.893		
Social influence (SI)	SI1	0.866	0.827	0.685	0.867
	SI2		0.803		
	SI3		0.853		
Perceived risk (PR)	PR1	0.892	0.809	0.736	0.893
	PR2		0.867		
	PR3		0.896		
Trust (TR)	TR1	0.913	0.834	0.782	0.915
	TR2		0.926		
	TR3		0.891		
Relative advantage (RA)	RA1	0.928	0.931	0.813	0.929
	RA2		0.899		
	RA3		0.874		
Intention to use (ITU)	ITU1	0.957	0.947	0.887	0.959
	ITU2		0.966		
	ITU3		0.912		

**TABLE 2:** Discriminant tests.

	*PE*	EE	SI	PR	TR	RA	ITU
PE	0.860						
EE	0.625	0.888					
SI	0.563	0.390	0.828				
PR	–0.084	–0.034	–0.292	0.858			
TR	0.567	0.472	0.582	–0.176	0.884		
RA	0.744	0.509	0.630	–0.171	0.598	0.902	
ITU	0.702	0.539	0.624	–0.280	0.543	0.745	0.942

*PE, performance expectancy; EE, effort expectancy; SI, social influence; PR, perceived risk; TR, trust; RA, relative advantage; ITU, intention to use.*

This research performed Harman’s one-factor test (Podsakoff and Organ, 1986), which has been adopted by Wagner and Charinsarn (2021) and Wagner et al. (2021) for evaluating common method variance (CMV). As a result, the most significant element explained 49.3% of the variance, which is smaller than the 50% threshold. Hence, the CMV was not a critical problem in this study.

### Model Fit

As reported in Table 3, fit indices of measurement and structural models satisfied the index guidelines (Hair et al., 2010).

**TABLE 3:** Fit indices.

Indices	Measurement model	Structural model	Recommendation
Chi-square/df	2.192	2.259	<3
Comparative fit indices	0.966	0.963	>0.9
Normed fit indices	0.939	0.935	>0.9
Root mean square error of approximation	0.063	0.064	<0.08
Incremental fit indices	0.966	0.963	>0.9

### Hypothesis Tests

**FIGURE 1:**
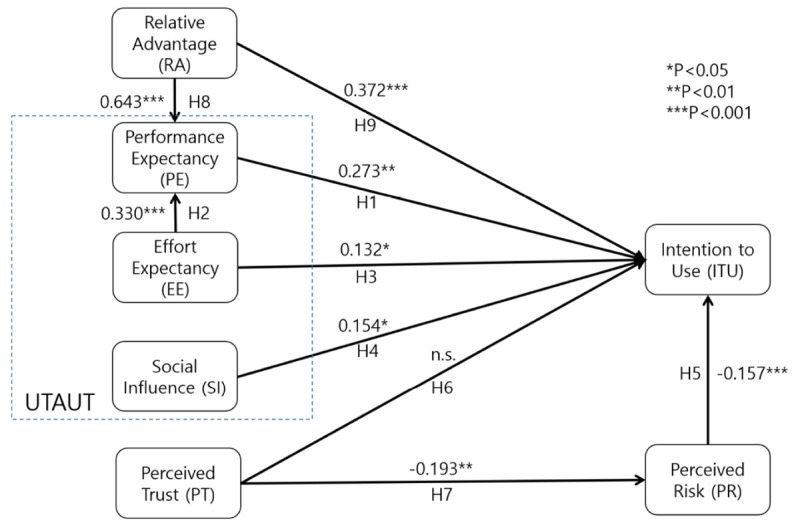
Hypothesized relationships.

According to the results of SEM (Figure 1), in this study, eight hypotheses were accepted while one was rejected (H6). Moreover, the antecedent factors resulted in 70.0% of the variance in intention. Specifically, relative advantage [H9, β = 0.372, SE = 0.095, critical ratio (CR) = 4.159], performance expectancy (H1, β = 0.273, SE = 0.108, CR = 2.975), effort expectancy (H3, β = 0.132, SE = 0.063, CR = 2.346), social influence (H4, β = 0.154, SE = 0.070, CR = 2.512), and perceived risk (H5, β = −0.157, SE = 0.038, CR = −4.173) all exerted significant impact on intention. In addition, relative advantage (H8, β = 0.643, SE = 0.048, CR = 12.102) and effort expectancy (H2, β = 0.330, SE = 0.047, CR = 6.644) also had a positive effect on performance expectancy. Furthermore, trust was found to exert a negative influence on perceived risk (H7, β = −0.193, SE = 0.078, CR = −3.102). However, the association between trust and intention (H6) was not supported.

## Discussion and Conclusion

This study was conducted to investigate the predictors that affected consumers’ intentions to use FRP in offline markets. Although the research model was constructed incorporating only six antecedent factors, it presented a relatively high percentage (70.0%) of the variance in consumers’ intentions, compared to other UTAUT-based empirical studies (Bhatiasevi, 2016; Madigan et al., 2017; Bawack and Kamdjoug, 2018; Patil et al., 2020; Wang et al., 2020). Moreover, its explanatory power is greater than that presented by Zhang and Kang (2019), who investigated FRP adoption. Thus, it can be concluded that extending the UTAUT-based model by employing relative advantage, perceived risk, and trust is an effective way for exploring the users’ intentions to use FRP. Furthermore, several meaningful implications can be obtained as follows.

### Theoretical Implications

First, the relative advantage was found to be the most important factor in FRP adoption. This may be due to the pervasive nature of Fintech services in China. As of 2020, there has been extensive use of various payment services (e.g., quick response code and fingerprint) in Chinese offline markets. For instance, the transactions’ size of offline quick response code payments reached 700 million Yuan in 2019 (Insight and Info, 2020). Thus, the consumers perceiving the advantages of FRP over existing payment services is a factor that strongly influences their intentions to use it. If the relative advantage of FRP is not understood, the probability of consumers using it instead of the existing payment services is very low. In conclusion, consumers are motivated to use FRP when they feel the relative advantage of FRP.

Second, the relative advantage was found to positively and strongly affect performance expectancy implying that consumers are expected to perceive FRP as being beneficial when its advantages are believed to be superior to that of existing payment services by the consumers. A few empirical studies support this notion as well [e.g., smart watch (Kim and Shin, 2015) and Uber mobile application (Min et al., 2019)]. However, this has rarely been demonstrated in the field of Fintech. Therefore, it is recommended that a combination of UTAUT and innovation diffusion theory-based model is used to consider this relationship and thus obtain a better understanding of innovative products and services (e.g., Fintech service, virtual reality game, and smart home).

Third, UTAUT constructs were observed to significantly affect intention in the positive direction, implying FRP will be used by consumers when they feel that using it is beneficial to them and does not require much effort. In addition, consumers are more likely to use FRP if they perceive people known to them using FRP. However, several earlier studies (e.g., Slade et al., 2015; Oliveira et al., 2016; Lee et al., 2019b) have demonstrated that effort expectancy does not significantly affect the consumers’ intentions to utilize specific Fintech services due to the consumers’ familiarity with using specific Fintech services (Hackbarth et al., 2003). Thus, based on this, it was concluded that as the FRP service is yet to be extensively used in China, most of the consumers are unfamiliar with its workings. Thus, consumers consider the ease of using FRP an important factor when attempting to use it. Consequentially, effort expectancy plays a notable role in FRP adoption as observed in this study.

Fourth, effort expectancy positively affects performance expectancy, i.e., consumers consider FRP as beneficial when they can use it effortlessly. This notion is supported by certain UTAUT-based research on Fintech service adoption (Oliveira et al., 2016; Alalwan et al., 2017). According to previous studies, the performance expectancy and the effort expectancy can also be considered as perceived usefulness and perceived ease of use, respectively (Venkatesh et al., 2003; Zhou et al., 2010). Moreover, TAM concurs that enhancing perceived ease of use induces greater perceived usefulness (Davis, 1989). Thus, this result is also supported by TAM and TAM-based empirical studies (Davis, 1989; Hassan and Wood, 2020). However, despite this relationship being theoretically sound, several UTAUT-based studies (Bhatiasevi, 2016; Rodrigues et al., 2016; Hoque and Sorwar, 2017; Madigan et al., 2017; Bawack and Kamdjoug, 2018; Cao and Niu, 2019; Patil et al., 2020) have not considered it while conducting the study. Thus, this study proposes that the connection between performance expectancy and effort expectancy should be considered when constructing UTAUT-based models. This is expected to aid in understanding the adoption of specific technologies (e.g., Fintech service).

Fifth, perceived risk was found to play a significant role in reducing intention. In contrast, trust did not affect the intention to use FRP. These results may be due to the following reasons. As of 2020, FRP is still in its initial stages of development, and thus, its security has not been evaluated for a long time. In addition, the consumers are required to provide their facial feature information to use FRP service, which may cause them to feel insecure in terms of their privacy (Garaus et al., 2021). Furthermore, news articles are increasingly focusing on the risks of FRP (South China Morning Post, 2019), which may motivate individuals to build a negative perspective of FRP (Brown and Deegan, 1998; Cao and Niu, 2019). Thus, the security of this service can be considered unstable by users. In this context, regardless of consumers trusting the service providers, they are likely to refuse using it due to the awareness of the risks associated with this service. Thus, the consumers’ perceived risk is an important factor that reduces intention, while trust does not exhibit a notable relationship with intention. This perspective is supported by Slade et al. (2015), who stated that when the new technology is considered highly risky, the perceived risk becomes a dominant factor influencing intention in comparison with the impact of trust.

Finally, the notable role of trust in reducing perceived risk as observed in this study is supported in previous studies as well. This implies that consumers’ anxiety and uncertainty toward FRP decrease when they feel that the FRP service providers are trustworthy. Overall, trust inversely affects perceived risk which in turn inversely affects intention to use, implying that trust indirectly and positively influences intention to use.

### Practical Implications

First, it was observed that relative advantage plays the most important role in enhancing FRP adoption. Therefore, service providers must mainly focus on enhancing consumers’ perceptions of FRP’s relative advantage. Companies can emphasize the functional strength of FRP service that surpasses existing payment services (e.g., quick response code payment). In addition, highlighting the relative advantage of FRP, such as the availability without mobile devices (e.g., smartphone) and the intuitiveness of the payment process, can be an effective advertising strategy for FRP.

Second, performance expectancy was found to strongly affect consumers’ intentions to use FRP. Therefore, to satisfy the consumers’ performance expectations, service providers must improve FRP service based on the demands of the consumers. In particular, due to the FRP service still not being extensively used in China, companies should focus on increasing the number of offline stores with FRP usage capability, to make the consumers feel the convenience and usefulness of this service. As a result, consumers’ performance expectations on FRP service can be increased.

Third, effort expectancy notably affects consumers’ intentions to use FRP in a positive direction, implying that how to use FRP service in detail can be introduced when promoting the service to consumers. This allows consumers to feel that using FRP does not require much effort.

Fourth, social influence can enhance consumers’ intentions to use FRP. Thus, companies should facilitate the consumers’ recommendation behaviors and viral marketing (Kalinic et al., 2019). For instance, social media platforms such as TikTok, one of the most popular social network services in recent years, can be used by companies for this purpose. Through ways to encourage TikTok users to use FRP and share their experiences on the platform, companies may effectively increase the user base.

Finally, perceived risk can inversely predict consumers’ intentions to use FRP, indicating the importance of reducing the perceived risk. This can be realized if service providers can increase consumers’ trust. For example, service providers must fulfill their promises and protect consumers from possible privacy violations (e.g., the violation of consumers’ facial feature data).

Furthermore, due to the development of face recognition technology still being in its nascent stages, the recognition accuracy and security of the FRP system must be continuously improved. This also can aid in reducing the consumers’ perceived risk toward FRP.

### Limitations and Future Studies

Although this study yielded several meaningful implications, there exist several limitations.

First, from our results, only 28% of participants had used FRP in offline stores. However, this is not a serious limitation. Moreover, it can reflect the current status of the Chinese Fintech market wherein most of the consumers have not used the FRP service because it gained momentum in the offline market in 2019. Therefore, this limitation does not undermine the contribution of this study. Moreover, as stated in prior studies (Anderson and Gerbing, 1988; Holbert and Stephenson, 2002), sample sizes smaller than 200 are not expected to be suitable for achieving valid SEM results. Thus, the difference in perception between people who have already used FRP and those who have not was not explored in this study.

Second, although the model in this study explained a relatively high percentage of variance in consumers’ intentions, other relationships must be incorporated in the model to explore their influence on FRP adoption. For instance, based on the guidelines of Mariani et al. (2021), examining whether an individual’s trust in the technical system significantly affects their intention to use FRP can be meaningful in future studies. In addition, the respondents’ demographic information can be considered in further studies as well. Several studies have reported that consumers’ demographic information can notably influence the adoption of specific services (Guo et al., 2015).

Finally, because the sample was only from China, future research on the adoption of FRP must test the results in the context of other countries or cultures. For example, the FRP service has been launched in 2019 by a Korean company “Shinhan Card” under the brand name “Face pay” (The Korea Times, 2020). Thus, exploring the key predictors of Korea’s adoption of Face pay can yield meaningful results.

## Data Availability Statement

The original contributions presented in the study are included in the article/supplementary material, further inquiries can be directed to the corresponding author/s.

## Ethics Statement

Ethical review and approval was not required for the study on human participants in accordance with the local legislation and institutional requirements. The patients/participants provided their written informed consent to participate in this study.

## Author Contributions

DN: research design, implementation, writing, and editing. YK: writing and editing. JH: implementation. HJ: writing. JK: research design, writing, and implementation. All authors contributed to the article and approved the submitted version.

## Conflict of Interest

The authors declare that the research was conducted in the absence of any commercial or financial relationships that could be construed as a potential conflict of interest.

## Publisher’s Note

All claims expressed in this article are solely those of the authors and do not necessarily represent those of their affiliated organizations, or those of the publisher, the editors and the reviewers. Any product that may be evaluated in this article, or claim that may be made by its manufacturer, is not guaranteed or endorsed by the publisher.

## Appendix

**TABLE 1:** Questionnaire items.

Constructs	Items
Performance expectancy	1. FRP service would make my payment convenient in offline store.
	2. FRP service would make my payment efficient in offline store.
	3. I think FRP service is useful.
Effort expectancy	1. Learning how to use FRP will not require much effort.
	2. I think using FRP is easy.
	3. It will be easy to become skillful at using FRP.
Social Influence	1. People around me think that I should use FRP in offline store.
	2. I would use FRP to make payments at an offline store because most of my friends or families make their payments using FRP at offline stores.
	3. People who make significant influence on me think that I should use FRP in offline store.
Intention to Use	1. If provided a chance, I will use FRP in offline store.
	2. If provided a chance, I am willing to use FRP in offline store.
	3. If provided a chance, I intend to use FRP in offline store.
Perceived risk	1. Using FRP would put my privacy at risk.
	2. There is a risk of losing money if I use FRP to make payments.
	3. I think that making payments at an offline store using FRP is risky.
Trust	1. I believe FRP service providers keep consumers’ interests in mind.
	2. FRP service providers are trustworthy.
	3. FRP service providers will do everything to protect consumers’ transactions.
Relative advantage	1. FRP service is more convenient than other payment services.
	2. FRP service is more efficient than other payment services.
	3. FRP service offers more advantages than other payment services.

## References

[B1] Abudarham N. Shkiller L. Yovel G. (2019). Critical features for face recognition. *Cognition* 182 73–83. 10.1016/j.cognition.2018.09.002 30218914

[B2] Alalwan A. A. Dwivedi Y. K. Rana N. P. (2017). Factors influencing adoption of mobile banking by Jordanian bank customers: Extending UTAUT2 with trust. *Int. J. Inform. Manag.* 37 99–110. 10.1016/j.ijinfomgt.2017.01.002

[B3] Al-Saedi K. Al-Emran M. Ramayah T. Abusham E. (2020). Developing a general extended UTAUT model for M-payment adoption. *Technol. Soc.* 62:101293. 10.1016/j.techsoc.2020.101293

[B4] Anderson J. C. Gerbing D. W. (1988). Structural equation modeling in practice: A review and recommended two-step approach. *Psychol. Bull.* 103:411. 10.1037/0033-2909.103.3.411

[B5] Baptista G. Oliveira T. (2015). Understanding mobile banking: The unified theory of acceptance and use of technology combined with cultural moderators. *Comput. Hum. Behav.* 50 418–430. 10.1016/j.chb.2015.04.024

[B6] Bawack R. E. Kamdjoug J. R. K. (2018). Adequacy of UTAUT in clinician adoption of health information systems in developing countries: The case of Cameroon. *Int. J. Med. Inform.* 109 15–22. 10.1016/j.ijmedinf.2017.10.016 29195701

[B7] Bhatiasevi V. (2016). An extended UTAUT model to explain the adoption of mobile banking. *Inform. Devel.* 32 799–814. 10.1177/0266666915570764

[B8] Brown N. Deegan C. (1998). The public disclosure of environmental performance information—a dual test of media agenda setting theory and legitimacy theory. *Account. Bus. Res.* 29 21–41. 10.1080/00014788.1998.9729564

[B9] Cao Q. Niu X. (2019). Integrating context-awareness and UTAUT to explain Alipay user adoption. *Int. J. Industr. Ergon.* 69 9–13. 10.1016/j.ergon.2018.09.004

[B10] Chang J. Lee D. (2022). Changes in user experience and satisfaction as media technology evolves: The reciprocal relationship between video games and video game-related media. *Technol. Forecast. Soc. Change* 174:121219. 10.1016/j.techfore.2021.121219

[B11] Chiyangwa T. B. Alexander P. T. (2016). Rapidly co-evolving technology adoption and diffusion models. *Telem. Inform.* 33 56–76. 10.1016/j.tele.2015.05.004

[B12] Davis F. D. (1989). Perceived usefulness, perceived ease of use, and user acceptance of information technology. *MIS Q.* 13 319–340. 10.2307/249008

[B13] Dong Y. Su H. Wu B. Li Z. Liu W. Zhang T. (2019). “Efficient decision-based black-box adversarial attacks on face recognition,” in *Proceedings of the IEEE Conference on Computer Vision and Pattern Recognition*, (Netherland: IEEE), 7714–7722. 10.1109/CVPR.2019.00790

[B14] Featherman M. S. Pavlou P. A. (2003). Predicting e-services adoption: a perceived risk facets perspective. *Int. J. Hum. Comput. Stud.* 59 451–474. 10.1016/S1071-5819(03)00111-3

[B15] Fornell C. Larcker D. F. (1981). Evaluating structural equation models with unobservable variables and measurement error. *J. Market. Res.* 18 39–50. 10.1177/002224378101800104

[B16] Garaus M. Wagner U. Rainer R. C. (2021). Emotional targeting using digital signage systems and facial recognition at the point-of-sale. *J. Bus. Res.* 131 747–762. 10.1016/j.jbusres.2020.10.065

[B17] Guo X. Han X. Zhang X. Dang Y. Chen C. (2015). Investigating m-health acceptance from a protection motivation theory perspective: gender and age differences. *Telemed. Health* 21 661–669. 10.1089/tmj.2014.0166 25919800

[B18] Hackbarth G. Grover V. Mun Y. Y. (2003). Computer playfulness and anxiety: positive and negative mediators of the system experience effect on perceived ease of use. *Inform. Manag.* 40 221–232. 10.1016/S0378-7206(02)00006-X

[B19] Hair J. Black W. Babin B. Anderson R. (2010). *Multivariate Data Analysis*, 7th Edn. New Jersey, NJ: Prentice-Hall.

[B20] Hassan H. E. Wood V. R. (2020). Does country culture influence consumers’ perceptions toward mobile banking? A comparison between Egypt and the United States. *Telem. Inform.* 46:101312. 10.1016/j.tele.2019.101312

[B21] Holbert R. L. Stephenson M. T. (2002). Structural equation modeling in the communication sciences, 1995–2000. *Hum. Commun. Res.* 28 531–551. 10.1111/j.1468-2958.2002.tb00822.x

[B22] Hoque R. Sorwar G. (2017). Understanding factors influencing the adoption of mHealth by the elderly: An extension of the UTAUT model. *Int. J. Med. Inform.* 101 75–84. 10.1016/j.ijmedinf.2017.02.002 28347450

[B23] Hwang I. J. Lee B. G. Kim K. Y. (2014). Information asymmetry, social networking site word of mouth, and mobility effects on social commerce in Korea. *Cyberpsychol. Behav. Soc. Netw.* 17 117–124. 10.1089/cyber.2012.0566 24355038

[B24] Insight and Info (2020). Available online at: https://baogao.chinabaogao.com/hulianwang/473629473629.html

[B25] Jarvenpaa S. L. Tractinsky N. Saarinen L. (1999). Consumer trust in an Internet store: A cross-cultural validation. *J. Comput. Med. Commun.* 5:JCMC526. 10.1111/j.1083-6101.1999.tb00337.x

[B26] Johnson V. L. Kiser A. Washington R. Torres R. (2018). Limitations to the rapid adoption of M-payment services: Understanding the impact of privacy risk on M-Payment services. *Comput. Hum. Behav.* 79 111–122. 10.1016/j.chb.2017.10.035

[B27] Kabra G. Ramesh A. Akhtar P. Dash M. K. (2017). Understanding behavioural intention to use information technology: Insights from humanitarian practitioners. *Telemat. Inform.* 34 1250–1261. 10.1016/j.tele.2017.05.010

[B28] Kalinic Z. Marinkovic V. Molinillo S. Liébana-Cabanillas F. (2019). A multi-analytical approach to peer-to-peer mobile payment acceptance prediction. *J. Retail. Consum. Serv.* 49 143–153. 10.1016/j.jretconser.2019.03.016

[B29] Khalilzadeh J. Ozturk A. B. Bilgihan A. (2017). Security-related factors in extended UTAUT model for NFC based mobile payment in the restaurant industry. *Comput. Hum. Behav.* 70 460–474. 10.1016/j.chb.2017.01.001

[B30] Kim G. Shin B. Lee H. G. (2009). Understanding dynamics between initial trust and usage intentions of mobile banking. *Inform. Syst. J.* 19 283–311. 10.1111/j.1365-2575.2007.00269.x

[B31] Kim J. H. Nan D. Kim Y. Park M. H. (2021). Computing the User Experience *via* Big Data Analysis: A Case of Uber Services. *CMC* 67 2819–2829. 10.32604/cmc.2021.014922

[B32] Kim Y. Nan D. Kim J. H. (2021). Exploration of the Relationships Among Narcissism, Life Satisfaction, and Loneliness of Instagram Users and the High-and Low-Level Features of Their Photographs. *Front. Psychol.* 12:707074. 10.3389/fpsyg.2021.707074 34512463 PMC8427304

[B33] Kim K. J. Shin D. H. (2015). An acceptance model for smart watches. *Int. Res.* 25 527–541. 10.1108/IntR-05-2014-0126

[B34] Lahasan B. Lutfi S. L. San-Segundo R. (2019). A survey on techniques to handle face recognition challenges: occlusion, single sample per subject and expression. *Artif. Intell. Rev.* 52 949–979. 10.1007/s10462-017-9578-y

[B35] Lee J. Ryu M. H. Lee D. (2019a). A study on the reciprocal relationship between user perception and retailer perception on platform-based mobile payment service. *J. Retail. Consum. Serv.* 48 7–15. 10.1016/j.jretconser.2019.01.007

[B36] Lee J. M. Lee B. Rha J. Y. (2019b). Determinants of mobile payment usage and the moderating effect of gender: Extending the UTAUT model with privacy risk. *Int. J. Electr. Comm. Stud.* 10 43–64. 10.7903/ijecs.1644

[B37] Lee J. Lee D. Park Y. Lee S. Ha T. (2019c). Autonomous vehicles can be shared, but a feeling of ownership is important: Examination of the influential factors for intention to use autonomous vehicles. *Emerg. Technol.* 107 411–422. 10.1016/j.trc.2019.08.020

[B38] Li J. Wang J. Wangh S. Zhou Y. (2019). Mobile payment with alipay: An application of extended technology acceptance model. *IEEE Access* 7 50380–50387. 10.1109/ACCESS.2019.2902905

[B39] Li D. Su Q. Deng L. Cai K. (2019). “3D Reconstruction of Face Image Authentication Technology in Electronic Transaction Authentication,” in *IEEE Sensors Journal.* (Netherland: IEEE). 10.1109/JSEN.2019.2958655

[B40] Liébana-Cabanillas F. Molinillo S. Ruiz-Montañez M. (2019). To use or not to use, that is the question: Analysis of the determining factors for using NFC mobile payment systems in public transportation. *Technol. Forecast. Soc. Change* 139 266–276. 10.1016/j.techfore.2018.11.012

[B41] Lien C. H. Cao Y. Zhou X. (2017). Service quality, satisfaction, stickiness, and usage intentions: An exploratory evaluation in the context of WeChat services. *Comput. Hum. Behav.* 68 403–410. 10.1016/j.chb.2016.11.061

[B42] Lin X. Wu R. Lim Y. T. Han J. Chen S. C. (2019). Understanding the Sustainable Usage Intention of Mobile Payment Technology in Korea: Cross-Countries Comparison of Chinese and Korean Users. *Sustainability* 11:5532. 10.3390/su11195532

[B43] Liu Y. L. Yan W. Hu B. (2021). Resistance to facial recognition payment in China: The influence of privacy-related factors. *Telecommun. Policy* 45:102155. 10.1016/j.telpol.2021.102155

[B44] Madigan R. Louw T. Wilbrink M. Schieben A. Merat N. (2017). What influences the decision to use automated public transport? Using UTAUT to understand public acceptance of automated road transport systems. *Transport. Res. Part F* 50 55–64. 10.1016/j.trf.2017.07.007

[B45] Mariani M. M. Styven M. E. Teulon F. (2021). Explaining the intention to use digital personal data stores: An empirical study. *Technol. Forec. Soc. Change* 166:120657. 10.1016/j.techfore.2021.120657

[B46] Min S. So K. K. F. Jeong M. (2019). Consumer adoption of the Uber mobile application: Insights from diffusion of innovation theory and technology acceptance model. *J. Travel Tourism Market.* 36 770–783. 10.1080/10548408.2018.1507866

[B47] Nan D. Kim Y. Park M. H. Kim J. H. (2020). What Motivates Users to Keep Using Social Mobile Payments? *Sustainability* 12:6878. 10.3390/su12176878

[B48] Nan D. Lee H. Kim Y. Kim J. H. (2022). My video game console is so cool! A coolness theory-based model for intention to use video game consoles. *Technol. Forec. Soc. Change* 176:121451. 10.1016/j.techfore.2021.121451 34980931 PMC8716313

[B49] NetEase (2019). *NetEase.* Available online at: https://www.163.com/dy/article/EPP8MO2T0511TKH3.html (accessed September 23, 2019).

[B50] NetEase (2021). *NetEase.* Available online at: https://www.163.com/dy/article/GB64AQ990518U8EU.html (accessed January 16, 2022).

[B51] Nunnally J. C. (1978). *Psychometric Theory: 2d Ed.* New York, NY: McGraw-Hill.

[B52] Oh J. Yoon S. J. (2014). Validation of haptic enabling technology acceptance model (HE-TAM): Integration of IDT and TAM. *Telematics Inform.* 31 585–596. 10.1016/j.tele.2014.01.002

[B53] Oliveira T. Thomas M. Baptista G. Campos F. (2016). Mobile payment: Understanding the determinants of customer adoption and intention to recommend the technology. *Comput. Hum. Behav.* 61 404–414. 10.1016/j.chb.2016.03.030

[B54] Patil P. Tamilmani K. Rana N. P. Raghavan V. (2020). Understanding consumer adoption of mobile payment in India: Extending Meta-UTAUT model with personal innovativeness, anxiety, trust, and grievance redressal. *Int. J. Inform. Manag.* 54:102144. 10.1016/j.ijinfomgt.2020.102144

[B55] Pavlou P. A. (2003). Consumer acceptance of electronic commerce: Integrating trust and risk with the technology acceptance model. *Int. J. Electr. Comm.* 7 101–134. 10.1080/10864415.2003.11044275

[B56] Phonthanukitithaworn C. Sellitto C. Fong M. W. L. (2015). User intentions to adopt mobile payment services: A study of early adopters in Thailand. *J. Internet Bank. Comm.* 20 1–29.

[B57] Podsakoff P. M. Organ D. W. (1986). Self-reports in organizational research: Problems and prospects. *J. Manag.* 12 531–544. 10.1177/014920638601200408

[B58] Rodrigues G. Sarabdeen J. Balasubramanian S. (2016). Factors that influence consumer adoption of e-government services in the UAE: A UTAUT model perspective. *J. Internet Comm.* 15 18–39. 10.1080/15332861.2015.1121460

[B59] Rogers E. M. (2003). *Diffusion of Innovations.* New York, NY: Free Press.

[B60] Rogers E. M. Shoemaker F. (1983). *Diffusion of Innovation: A Cross-Cultural Approach.* New York, NY: Free Press.

[B61] Shao Z. Zhang L. Li X. Guo Y. (2019). Antecedents of trust and continuance intention in mobile payment platforms: The moderating effect of gender. *Electr. Comm. Res. Appl.* 33:100823. 10.1016/j.elerap.2018.100823

[B62] Shaw N. Sergueeva K. (2018). The non-monetary benefits of mobile commerce: Extending UTAUT2 with perceived value. *Int. J. Inform. Manag.* 45 44–55. 10.1016/j.ijinfomgt.2018.10.024

[B63] Shin D. H. (2009). Towards an understanding of the consumer acceptance of mobile wallet. *Comput. Hum. Behav.* 25 1343–1354. 10.1016/j.chb.2009.06.001

[B64] Slade E. L. Dwivedi Y. K. Piercy N. C. Williams M. D. (2015). Modeling consumers’ adoption intentions of remote mobile payments in the United Kingdom: extending UTAUT with innovativeness, risk, and trust. *Psychol. Market.* 32 860–873. 10.1002/mar.20823

[B65] Sohn K. Kwon O. (2020). Technology acceptance theories and factors influencing artificial Intelligence-based intelligent products. *Telem. Inform.* 47:101324. 10.1016/j.tele.2019.101324

[B66] South China Morning Post (2019). *China’s Subways Embrace Facial Recognition Payment Systems Despite Rising Privacy Concerns.* Available online at: https://www.scmp.com/tech/apps-social/article/3040398/chinas-subways-embrace-facial-recognition-payment-systems-despite (accessed June 20, 2021).

[B67] Statista (2021a). *Population Distribution in China in 2020, by Five-Year Age Group.* Available online at: https://www.statista.com/statistics/1101677/population-distribution-by-detailed-age-group-in-china/ (accessed January 16, 2022).

[B68] Statista (2021b). *Total Population of China from 1980 to 2020 with Forecasts Until 2026.* Available online at: https://www.statista.com/statistics/263765/total-population-of-china/ (accessed January 16, 2022).

[B69] The Korea Times (2020). *Shinhan Card Launches ‘Face Pay’ Service for 1st time in Korea.* Available online at: https://www.koreatimes.co.kr/www/biz/2020/07/175_287621.html (accessed June 20, 2021).

[B70] The Moodie Davitt Report (2019). *Shinsegae first: WeChat Pay facial recognition payments introduced outside Greater China.* Available online at: https://www.moodiedavittreport.com/shinsegae-first-wechat-pay-facial-recognition-payments-introduced-outside-greater-china/ (accessed June 20, 2021).

[B71] Venkatesh V. Morris M. G. Davis G. B. Davis F. D. (2003). User acceptance of information technology: Toward a unified view. *MIS Q.* 27 425–478. 10.2307/30036540

[B72] Wagner U. Charinsarn A. R. (2021). What language should be displayed on product packaging? How unconventional lettering influences packaging and product evaluation. *J. Int. Consum. Market.* 33 1–18. 10.1080/08961530.2020.1741483

[B73] Wagner U. Jacob I. Khanna M. Rai K. A. (2021). Possession Attachment toward Global Brands: How the “World of Barbie” is Shaping the Mindsets of Millennial Girls. *J. Int. Consum. Market.* 33 434–451. 10.1080/08961530.2020.1813671

[B74] Wang H. Tao D. Yu N. Qu X. (2020). Understanding consumer acceptance of healthcare wearable devices: An integrated model of UTAUT and TTF. *Int. J. Med. Inform.* 139:104156. 10.1016/j.ijmedinf.2020.104156 32387819

[B75] Warshaw P. R. Davis F. D. (1985). Disentangling Behavioral Intention and Behavioral Expectation. *J. Exp. Soc. Psychol.* 21 213–228. 10.1016/0022-1031(85)90017-4

[B76] Yuen K. F. Wang X. Ng L. T. W. Wong Y. D. (2018). An investigation of customers’ intention to use self-collection services for last-mile delivery. *Transport Policy* 66 1–8. 10.1016/j.tranpol.2018.03.001

[B77] Zhang S. Wang X. Liu A. Zhao C. Wan J. Escalera S. (2019). “A dataset and benchmark for large-scale multi-modal face anti-spoofing,” in *Proceedings of the IEEE Conference on Computer Vision and Pattern Recognition*, (Netherland: IEEE), 919–928. 10.1109/CVPR.2019.00101

[B78] Zhang W. K. Kang M. J. (2019). Factors affecting the use of facial-recognition payment: An example of Chinese consumers. *IEEE Access* 7 154360–154374. 10.1109/ACCESS.2019.2927705

[B79] Zhao A. L. Koenig-Lewis N. Hanmer-Lloyd S. Ward P. (2010). Adoption of internet banking services in China: is it all about trust? *Int. J. Bank Market.* 28 7–26. 10.1108/02652321011013562

[B80] Zhi H. Liu S. (2019). Face recognition based on genetic algorithm. *J. Vis. Commun. Image Represent.* 58 495–502. 10.1016/j.jvcir.2018.12.012

[B81] Zhong Y. Oh S. Moon H. C. (2021). Service transformation under industry 4.0: Investigating acceptance of facial recognition payment through an extended technology acceptance model. *Technol. Soc.* 64:101515. 10.1016/j.techsoc.2020.101515

[B82] Zhou T. (2012). Examining mobile banking user adoption from the perspectives of trust and flow experience. *Inform. Technol. Manag.* 13 27–37. 10.1007/s10799-011-0111-8

[B83] Zhou T. Lu Y. Wang B. (2010). Integrating TTF and UTAUT to explain mobile banking user adoption. *Comput. Hum. Behav.* 26 760–767. 10.1016/j.chb.2010.01.013

